# Supplementary material for: Learning curve and surgical outcome of robotic assisted colorectal surgery with ERAS program
Source: Sci Rep. 2022 Nov 29;12:20566. doi: 10.1038/s41598-022-24665-w (PMC9709162; doi:10.1038/s41598-022-24665-w)
Supplement: Supplementary file 1 — Supplementary Information 1. [file 41598_2022_24665_MOESM1_ESM.zip › LSC Raw data-Ñ[▒K/143/N_ñw╝╨░O▒Kñσ.pdf]

W83 / W83

臺中榮民總醫院
手術室護理記錄

[redacted]

基本資料

- 室別： 二醫手術室-35室，手術科別： CRS，手術類別： 常規，日期： 2021/10/25
- 進入時間： 1330 ； 麻醉開始時間： 1335 ； Time Out時間： 1458 ； 劃刀時間： 1459 ； 手術結束時間： 1755 送出時間： 1815 ；
- 麻醉方式： GE
  診斷：Malignant tumor of sigmoid colon 20CM FAV
  手術名稱：Laparoscopic Anterior resection and anastomosis - malignant
  術前預防性抗生素：
  - 抗生素名稱　：Veterin 1GM IVD 由麻醉護理師給予
  - 時間： 1450
  - 上次簽章: 賴思羽

  ，追加預防性抗生素：
- 手術醫師： 蔣鋒帆
- 麻醉醫師： VS 張思玲，麻醉醫師： R 魯成旭
-
  - 刷手護士A ： 顏育?，時間： 1330
  - 流動護士A ： 謝孟君，時間： 1330，接替流動一： 賴思羽，時間： 1500，接替流動二： 顏育銹，時間： 1800

皮膚狀況

A-V Shunt： 無
術前： 完整
　記錄者： 上次簽章: 賴思羽
術後： 完整
　記錄者： 顏育？

手術準備

手術臥式 單一手術臥式

-
  - 手術臥式： 截石臥式
  - 使用附件： 溫毯 38 ℃，頭圈，腳架，其它： 頭架
  - 皮膚準備： 10%Aq Betadine，2% Chlorhexidine
  - 電燒： 使用電燒: 電極片位置： 右大腿，記錄者： 上次簽章: 賴思羽

止血帶T： 無

植入物

- 植入物名稱/號碼/數量/部位：Surgiwrap 100mm x 130mm/1片/COLON

，記錄者： 上次簽章: 賴思羽

標本送檢

- 細菌培養 無
- 細胞學 無
- 冰凍切片 無
- 病理標本
  組織名稱一：IMA Root
  ,
  組織名稱二：Distal cut end
  ,
  組織名稱三：S-colon
- 截肢 無

標本交付 標本： 3 袋，標本： 0 盒，標本： 0 瓶，細胞學： 0 管，Culture Tube： 0 管，被交付醫師： Fellow 林陳彥，

- 記錄者： 上次簽章: 賴思羽
- 時間： 1745

傷口術後敷料

傷口敷料： 傷口敷料： 無

- 請於圖上標示部位：

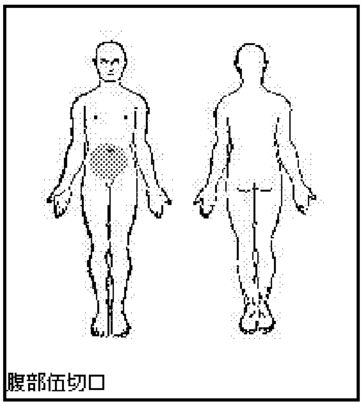

- 傷口敷料： 傷口凝膠+MEPORE

導管

導管	天數	導管資訊	插入日期	插入時間	更換日期	拔除日期	拔除時間	拔除原因	預計拔除/更換	預計拔除/更換日期
Double Lumen CVC(1)	1	號碼:8 Fr; 部位:; 象限:RU; 註記:15公分;	2021/10/25	1420						
Pig-tail（chest）(1)	1	號碼:16 Fr.; 部位:左側胸腔; 註記:固定17cm;	2021/10/25	1415						
Foley(1)	1	號碼:16 Fr.; 部位:膀胱; 註記:Balloon 10ml;	2021/10/25	1430						
A-line(1)	1	號碼:22G; 部位:左橈動脈;	2021/10/25	1430						

用物計數

- 手術中出血量： MINIMAL c.c.
- 手術中尿量： 150 c.c.
-
    - 其它： 無

用物計數： 多切口同時縫合

- 1.器械： 正確，2.縫針： 正確，3.刀片： 正確，4.紗布： 正確，5.腹部墊： 未使用，6.紗球： 未使用，7.PEANUTS ： 未使用，8.紗條： 未使用，9.絲帶： 未使用，10.針頭： 未使用，11.Vessel loop： 未使用，12.Boots： 未使用，13.Jelco： 未使用，14.棉條： 未使用，
    - 第一次：
        - 刷手護士 ： 顏育銹
        - 流動護士： 上次簽章: 賴思羽
        - 時間： 1720

    ,
    - 第二次：
        - 刷手護士 ： 顏育銹
        - 流動護士： 上次簽章: 賴思羽
        - 時間： 1730

    ,
    - 第三次：
        - 刷手護士 ： 顏育銹
        - 流動護士： 上次簽章: 賴思羽
        - 時間： 1740

備註

備註： NIL

- 記錄者： 顏育？ 上次簽章: 賴思羽
- 時間： 1820

填寫護理人員 顏育？

正式摘要

W83 / W83

doctorRank:B
